# The addition of anlotinib reduces the incidence of radiation and immunotherapy-induced pneumonia

**DOI:** 10.1186/s12967-023-04500-0

**Published:** 2023-10-01

**Authors:** Linlin Yang, Qian Zhao, Jinming Yu, Linlin Wang

**Affiliations:** 1 Department of Oncology, Renmin Hospital of Wuhan University, Wuhan, 430064 China; 2 Department of Radiation Oncology and Shandong Provincial Key Laboratory of Radiation Oncology, Shandong First Medical University and Shandong Academy of Medical Sciences, Jinan, Shandong China; 3 Research Unit of Radiation Oncology, Chinese Academy of Medical Sciences, Jinan, Shandong China

## To the editor,

The synergistic effect of immunotherapy and radiotherapy has been widely recognized. However, the cumulative lung toxicity of thoracic radiotherapy (TRT) combined with PD-1/PD-L1 inhibitors cannot be overlooked in clinical practice. In the PACIFIC study, the incidence of pneumonitis was significantly higher in the immunotherapy consolidation group compared to the placebo group (33.9% vs. 24.8%) [1]. However, in the IMpower150 study, the addition of bevacizumab (ABCP group) resulted in a lower incidence of pneumonitis compared to the ACP group, and the resolution of inflammation was better (4.3% vs. 2.8%, 29.4% vs. 9.1%) [2]. Preclinical studies have also shown that anti-vascular endothelial growth factor antibodies could prevent pulmonary alveolar leakage caused by increased vascular permeability, thereby alleviating immunotherapy-induced pneumonitis [3]. Therefore, we hypothesize that anti-angiogenic drugs may reduce the incidence of pneumonitis caused by the combination of radiotherapy and immunotherapy.

The study was approved by the Ethics Committee of Shandong Cancer Hospital. As it was a retrospective analysis, informed consent was not required. We included patients with non-small cell lung cancer (NSCLC) who received TRT and at least 2 cycles of immunotherapy, with the interval between the two treatments not exceeding 6 months. The primary endpoint was treatment-related pulmonary toxicity, assessed using the Common Terminology Criteria for Adverse Events (version 5.0).

A total of 148 patients who received thoracic conventional fractionated radiation therapy were included (Table 1), with a median total radiation dose of 54.7 Gy (range 30–66 Gy). Among them, 72 cases (48.6%) experienced pneumonitis, and 42 cases (28.3%) developed grade ≥ 2 pneumonitis. Previously, 43 patients (29.0%) received anti-angiogenic treatment during radiotherapy or immunotherapy, with 14 cases (9.4%) receiving bevacizumab and 29 cases (19.5%) receiving anlotinib.

**Table 1:** Clinical characteristics of patients

Characteristics	Patients, No. (%)		P
	Grade < 2 pneumonitis (N = 106)	Grade ≥ 2 pneumonitis (N = 42)	
*Age*			0.311
≤ 65	70 (66.0%)	24 (57.1%)	
> 65	36 (34.0%)	18 (42.9%)	
*Sex*			0.594
Male	87 (82.1%)	36 (85.7%)	
Female	19 (17.9%)	6 (14.3%)	
*Smoke*			0.921
Smoker	47 (44.3%)	19 (45.2%)	
Non-smoker	59 (55.7%)	23 (54.8%)	
*KPS*			0.991
< 80	5 (4.7%)	2 (4.8%)	
≥ 80	101 (95.3%)	40 (95.2%)	
*Stage*			0.638
I–II	2 (1.9%)	2 (4.8%)	
III	63 (59.4%)	25 (59.5%)	
IV	41 (38.7%)	15 (35.7%)	
*Histology*			0.254
Adenocarcinoma	50 (47.2%)	20 (47.6%)	
Squamous cell carcinoma	52 (49.1%)	22 (52.4%)	
Other	4 (3.8%)	0 (0.0%)	
*ICIs + TRT*			0.259
Concurrent	17 (14.3%)	2 (24.1%)	
Sequential	102 (85.7%)	22 (75.9%)	
*RT Dose*			0.991
< 50	16 (15.1%)	6 (14.3%)	
50–60	42 (39.6%)	17 (40.5%)	
≥ 60	48 (45.3%)	19 (45.2%)	
*Anlotinib*			0.016
Yes	26 (24.5%)	3 (7.1%)	
No	80 (75.5%)	39 (92.9%)	
*Bevacizumab*			0.341
Yes	8 (7.5%)	6 (14.3%)	
No	98 (92.5%)	36 (85.7%)	

**Fig. 1:**
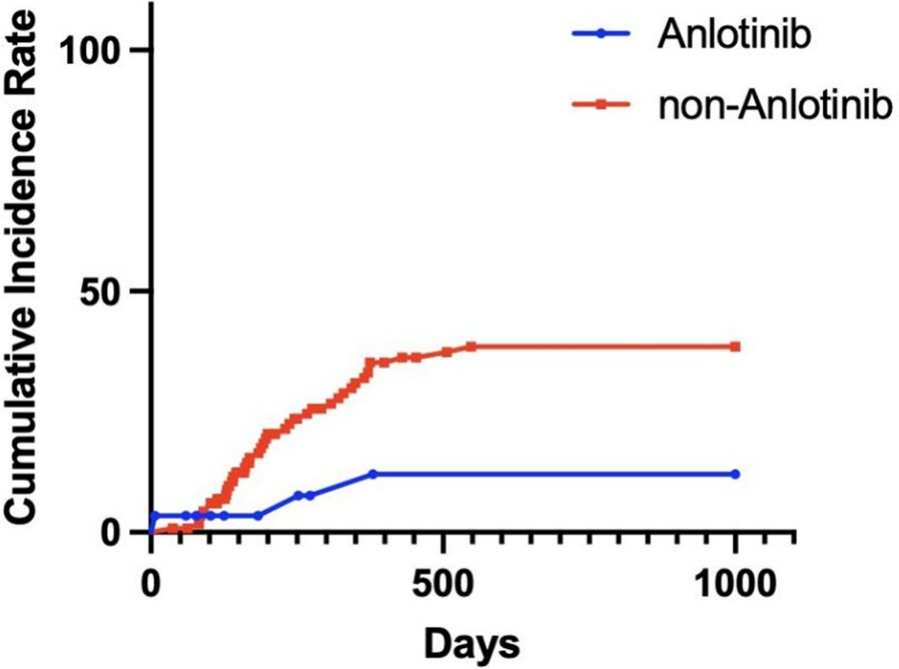
Cumulative incidence of grade ≥ 2 pneumonitis

Logistic regression analysis showed that prior treatment with anlotinib was a protective factor for pneumonitis compared to those who did not receive anlotinib. The incidence of pneumonitis was 31% vs. 52.9% (p = 0.038, HR = 0.400, 95% CI 0.168–0.950), and the incidence of grade ≥ 2 pneumonitis was 10.3% vs. 32.7% (p = 0.024, HR = 0.237, 95% CI 0.067–0.830) in the anlotinib-treated group and the non-anlotinib-treated group (Fig. 1), respectively. The incidence of grade ≥ 3 pneumonitis was 10.3% vs. 20.1% (p = 0.110, HR = 0.293, 95% CI 0.065–1.320). Gender, age, smoking index, prior treatment with bevacizumab, and total TRT dose were not identified as risk factors for the occurrence of pneumonia. Meanwhile, the addition of anlotinib did not increase the occurrence of other grade 3 or higher toxicities.

With the combined immunotherapy and TRT has become the standard treatment for NSCLC, the overlapping pulmonary toxicity of these two treatments has emerged as a common and severe complication.

Previous preclinical studies have shown that bevacizumab can alleviate immunotherapy-induced pneumonitis. Additionally, the IMpower150 study also found that the addition of bevacizumab resulted in a lower incidence of pneumonitis. However, in this current study, the application of bevacizumab did not show a lower incidence of pneumonitis. Instead, a multi-targeted small molecule anti-angiogenic drug, anlotinib, provided a surprising result, reducing the occurrence of pneumonitis caused by the combination of TRT and immunotherapy (p = 0.038, HR = 0.400, 95% CI 0.168–0.950). Nevertheless, the mechanisms behind this effect still require further investigation.

Currently, corticosteroids are commonly used in clinical practice to treat radiation-immunotherapy-related pneumonitis, but they may affect the anti-tumor efficacy of drugs [4]. On the other hand, anlotinib has been reported to exhibit synergistic effects with immunotherapy and to reverse radiotherapy resistance in NSCLC [5]. Our research results indicate that anlotinib may be a favorable treatment option for patients receiving combined radiotherapy and immunotherapy.

This study offers a new alternative for clinical treatment approaches; however, it necessitates larger prospective studies to explore safety and efficacy aspects further.

## Data Availability

The data that support the findings of this study are available from the corresponding author, Linlin Wang, upon reasonable request.
